# Learnings from the Adult Religious Education and Faith Development (AREFD) project for initial teacher education of religious educators

**DOI:** 10.1007/s40839-021-00152-8

**Published:** 2021-09-25

**Authors:** Bernadette Sweetman

**Affiliations:** Mater Dei Centre for Catholic Education (MDCCE), Dublin City University, St. Patrick’s Campus, Drumcondra, Dublin 9, D09 DY00 Ireland

**Keywords:** Adult religious education, Initial teacher education, Ireland, Lifelong learning

## Abstract

Since October 2018, researchers at the Mater Dei Centre for Catholic Education at Dublin City University have been engaged in the Adult Religious Education and Faith Development (AREFD) project. The overarching aim of the project was to facilitate a re-energising of adult religious education and faith development in Ireland. Working amongst local faith communities with an academic research focus, an area of interest that has emerged is how the insights gained from AREFD project can contribute to initial teacher education, particularly involving students preparing for employment as post-primary religious educators. This paper will outline some of the key themes that emerged from the data gathered in phase two of the AREFD project as it pertains to the initial teacher education (ITE) of religious educators. In phase two, a total of fourteen semi-structured interviews/focus groups were conducted between December 2019 and April 2021, featuring twenty-two people from across the island of Ireland who have a wealth of experience in AREFD across diverse contexts. The purpose of these interviews was to gather together the rich insights from the depth of experience of the interviewees on practicalities and possibilities central to adult religious education. The contexts in which they have worked are all pertinent to both the post-primary Religious Education curriculum in the Republic of Ireland and wider related learning experiences, in Ireland and beyond. Four key findings from this phase of the AREFD project are reported upon in this paper: the specific realm of AREFD as distinct from school-based religious education and catechesis; the need for intentional investment in AREFD; the physicality of religion; collaboration, communication and connection. These findings may contribute to the reflections of and course development by initial teacher education providers as they seek to offer the highest quality opportunities to their students, in the understanding that their students are adults themselves and that education is a lifelong endeavour.

## Introduction

The Adult Religious Education and Faith Development project (AREFD) began in October 2018 at the Mater Dei Centre for Catholic Education (MDCCE) at Dublin City University. Funded by the Presentation Sisters North East Province, the AREFD project has both pastoral and academic dimensions. The pastoral dimension is guided by the principles underpinning the Irish Episcopal Conference’s directory for catechesis which was published in 2010. This directory emerged as the local Catholic Church in Ireland’s response to the *General Directory for Catechesis* (Congregation for the Clergy, 1997). As multiculturalism, globalisation, and technological innovations continue to develop, the Church has a significant role to play in showing a way forward for its members in these changing and often challenging times. In *Share the Good News: National directory for catechesis in Ireland* ([SGN], Irish Episcopal Conference, 2010) the Catholic Church in Ireland places an emphasis on the centrality of adult faith development. It affirms the primary importance of adult faith development in facilitating members of the Church to grow into the fullness of lived Christian faith (SGN, paras. 68–90). In SGN, the Irish Episcopal Conference uses ‘faith development’ as an overarching term, ‘to encapsulate all the different approaches to ongoing education available to people from the beginning of their journey into Christian faith and throughout a lifetime of growth in that faith.’ (SGN, para. 43). It refers to the variety of terms used in association with Christian initiation, catechesis, and theological reflection. It also, of course, includes religious education. Religious education is the connecting point between the pastoral and academic dimensions of the AREFD project.

The Mater Dei Centre for Catholic Education (MDCCE) is one of two denominational centres of Dublin City University (DCU). It works closely with the newest faculty of the university, the Institute of Education. The Institute was formed in 2016 when four colleges were incorporated into DCU. Offering a variety of undergraduate and postgraduate courses in different disciplines, their key offering was initial teacher education. At primary level, these were St. Patrick’s College, Drumcondra, which espoused a Catholic ethos, and the Church of Ireland College of Education, which served the Irish Protestant community. At post-primary level, the Mater Dei Institute of Education, under Catholic patronage, provided initial teacher education in the curriculum area of Religious Education and other related areas. Each with a distinctive and highly regarded tradition, these three colleges merged with the School of Education Studies, already established at DCU to form the Institute of Education. This historic development brought together not only a rich diversity of traditions but also a highly respected staff community, both past and present, whose expertise in education has global renown. At the Institute of Education, initial teacher education is provided for early childhood, primary, and post-primary levels as well as further and higher education. It was envisaged at MDCCE the AREFD project could be a significant contribution to the university in the further development of the field of adult education.

The project with its ‘adult’, ‘religious education’ and ‘faith development’ components brings each of these three spheres together. The ongoing research can add new and creative opportunities for each of the different spheres to learn from each other, although insights gained from the project can be applied to many contexts, educational settings and age groups. Elsewhere, there have been initial accounts published of selected findings of the research up to this point (Byrne & Sweetman, 2021; Sweetman, 2019a, 2019b, 2021). This paper will focus on what can be learned from the AREFD project for those involved in the initial teacher education of religious educators, specifically those who aim to work at post-primary level in Catholic schools. It is therefore necessary to provide the reader with some background information. This includes a brief overview of the AREFD project; the nature of religious education at post-primary level in Ireland, and the theoretical rationale for applying learnings from the AREFD project to initial teacher education (ITE) of post-primary religious educators.

## Overview of the Adult Religious Education and Faith Development Project (AREFD)

Launched in October 2018, this was initially a three-year project. Due to the impact of COVID-19, it has been extended to December 2022. Broadly speaking, the project was created to facilitate a re-energising of adult religious education and faith development in Ireland. In the first year, an extensive literature review was conducted. Then, using a nationwide online survey, the research team carried out a scoping exercise to ascertain the different forms of AREFD taking place in Ireland and to identify key individuals and groups involved. The survey ran for six weeks in May/June 2019. It invited adults to explore their understanding and experiences of religious education when they were at school but also bring them beyond that, to what matters to them in the present day. It encouraged adults to reflect on how they express their beliefs and values; the opportunities (or lack thereof) for religious education/faith development at various stages of life; and, ultimately, what would Irish adults like to see happening in the future to engage them in ongoing religious education and faith development. From an early stage in the project, the research team became aware of two significant issues. Firstly, there was a lacuna in the corpus of literature on adult religious education and faith development in Ireland that combines the academic and pastoral. Secondly, there was a high level of interest in and desire for adult religious education and faith development opportunities in Ireland.

In conducting the literature review, it was initially noted that the majority of research and academic publications on religious education and faith development in the Irish context focused on the school environment and the transmission of faith to the next generations (Sweetman, 2021). The academic literature on adult education, in Ireland and elsewhere, more often looked at issues such as socio-economic, cultural and gender-related aspects to adult education. Adult religious education and faith development featured in literature from faith communities. This was more pastoral and practical than academic in nature. Where there was evidence of academic study in adult religious education, there was a tendency to focus on formal education, most commonly associated with training for ministry (Codd, 2017) or lay theological education (Elias, 2006).

Though the responses from the survey provided great insights and prompts for further investigation, it was a different and unexpected but welcome output from this phase of the project that impacted greatly upon the research team and the future direction of the study. This was the prompting of conversation about AREFD and a capturing of interest, amongst both pastoral and academic groups, in Ireland and beyond. While the total number of respondents to the survey was 738, meaning the sample could not be seen as representative of the entire Irish adult population, there were, however, respondents from every age group and every county. A strong level of engagement between the research team and various media channels was evident with a range of articles in print media, online and radio interviews. The research team received a number of enquiries from different sources during and after the survey, indicating that the project was quickly gaining momentum nationwide and beyond. It was soon clear that there was an appetite for meaningful conversations about adult religious education and faith development, both in academic and pastoral spheres. There was a sense that the AREFD project could provide a space in which the desire for such discourse on AREFD could contribute to the perceived gap in the academic literature, and that this convergence could be mutually beneficial to both the academic and pastoral spheres.

## Religious education in Ireland: examining the term using the example of post-primary Religious Education

The term ‘religious education’ is most commonly associated with the formal curriculum subject offered in schools. At post-primary level in the Republic of Ireland, Religious Education has been part of the state curriculum at Junior Cycle level (12- to 15-year olds) since 2000. In 2003, it was introduced at Senior Cycle level (16- to 18-year olds). In 2019, a new *Junior Cycle Religious Education Specification* (NCCA, 2019) was established as part of the overall revision of all subjects at this level in the *Framework for Junior Cycle 2015* (Department of Education and Skills, 2015). A review of Senior Cycle subjects is currently underway. The state-sponsored provision for Religious Education at post-primary level is part of the overall response of the Irish education sector to best equip our young people for all aspects of their future, both as individuals and members of society. The rationale for the *Junior Cycle Religious Education Specification* explains, ‘it facilitates the intellectual, social, emotional, spiritual and moral development of students’ (NCCA, 2019, p. 6). The specification is underpinned by the acknowledgement that growth in multiculturalism, plurality of faiths and other worldviews, globalisation, and technological advancements must be catered for in educational provisions today (Byrne, 2018). A study conducted amongst 13- to 15- year olds in the Republic of Ireland between 2013 and 2015, reported on in Byrne and Francis (2019), indicated a very positive response from the 3000 young people surveyed regarding the impact of their Religious Education on their lives (Byrne et al., 2019a, 2019b). Explaining in detail the rationale for and contribution of Religious Education in post-primary education is not a task for this paper, but it is documented elsewhere (Byrne, 2017, 2018, 2021; Catholic Schools Partnership, 2014; Hession, 2015; McGrady, 2014) For this paper, a broad understanding of religious education is needed. Religious education, though most associated with the subject, is much more expansive. The familiar formal settings such as that of schools, seminaries and other institutions are only part of the picture. Homes, communities, places of worship, experiences on holidays or pilgrimages, on the Internet, and engaging in group activities or with mentors are all contexts in which religious education can take place (Chazan, 2003; English, 2000; Simojoki, 2019). This is informal religious education and can apply to all groups, from young children to older adults (Byrne, 2008). Commenting particularly on the adult setting, Elias states that ‘the most pervasive adult religious education taking place today is informal education’ (2012, p. 9). His statement, however, also applies to other age groups.

## Rationale for applying learnings from the AREFD project to ITE of religious educators

A broad understanding of religious education as having formal and informal and non-formal aspects is complemented by an appreciation of education in general as a lifelong endeavour.

To be human means to learn. To be fully human entails a lifelong effort in acquiring knowledge, attitudes, skills and behaviours. The complexity of life and the constant changes that persons face, increasingly demand that adults continue to learn through their lives.

(Elias, 1993, p. 93)

Over this lifetime, our needs change as do our capacities to critically reflect, to make decisions, to open ourselves to new opportunities and to impact upon the lives of others. Our current generation of religious educators were once the school-going population. Our elderly friends and neighbours have gained a lifetime of experience and wisdom. The cycle of life goes on. With an appreciation of learning as lifelong, we also must consider the importance of relationships. The theory of education as relational is established, notably in the work of German philosopher Martin Buber (1947) and later developed further by Polish educator and paediatrician Janusz Korczak. A valuable exploration of the theories of both can be found in Boschki (2005), where he explains how both thinkers envisage relationship as a core principle in all education, not just religious education. The ‘I and Thou’ that features throughout the work of Buber in his treatise of education as relational and dialogical can be applied to the image of educator and learner. Complemented by Korczak’s pedagogy of equality (Boschki, 2005), we are further prompted to acknowledge that all parties in the educational encounter can learn from and be changed by the other. Students and teachers learn from each other. It is a mutual transaction, not a didactic one-way system. In this vein, it is proposed that educational systems can also mutually learn from each other. Educational providers in formal settings can (and should be open to) learn from those in informal and non-formal settings and vice versa. Similarly, those involved in early childhood, primary, post-primary, further, higher and adult education, can (and should be open to) learn from each other. ITE must respond to cultural and societal challenge, and maximise upon opportunities emerging from developments in educational theory, methodologies and other related areas. It is in this framework that four findings from the second phase of AREFD project are offered for consideration by those involved in the initial teacher education of religious educators.

## Method

The second phase of the AREFD project was to consult with a diverse sample of individuals and groups involved in the area. By drawing on their wisdom and experience, the research seeks to contribute, both at an academic and pastoral level, to the development of new AREFD opportunities.

## Procedure

The purpose of these interviews was to gather together the rich insights from the wealth of experience of the interviewees on the practicalities and possibilities central to adult religious education. The broad approach of an experiential mode of qualitative research (Braun & Clarke, 2013) was therefore adopted with a list of open-end questions constructed by the research team. Open-ended questions in a semi-structured interview style was the chosen data collection methodology with the aim of encouraging the participants to lead the discussion. The questions served as prompts for the participants to indicate the nature of their involvement in AREFD either as a provider and/or participant. In particular, the strengths and weaknesses of any particular activities were to be elicited. Whilst gathering accounts of the breadth of experience in AREFD among the interviewees, the research team also adopted a critical approach to the research and invited the participants to reflect upon and critique their experiences with a view to making recommendations for the future development of AREFD. All participants were provided with plain language statements and informed consent forms in keeping with the protocol required by the DCU Research Ethics Committee. In-person interviews were audio-recorded. In the instance of interviews conducted via Zoom during COVID-19 restrictions, permission was sought to record the consultation according to the DCU Data Protection Unit protocols on conducting research online. All consultations took place between December 2019 and April 2021.

## Participants

A purposive sampling strategy was employed to identify potential participants. The research team sought to engage with different forms of AREFD in a variety of contexts. Context in this case referred to the size of the projects in which participants were involved, their geographical location i.e. local/nationwide, and/or rural/urban, and the overall mode or genre of the AREFD taking place. Cognisant of the timeframe of the project and the capacity of the research team, a smaller number of participants from as diverse a range of contexts and geographical locations was preferable. Some participants had voluntarily contacted the research team in the earlier phase of the AREFD project expressing their interest to contribute in some way where possible. Other participants were approached where it was considered by the researchers that their context would complement the existing sample. Twenty-two people working in adult religious education and faith development, in both the Republic of Ireland and Northern Ireland and across a variety of settings were consulted in total in this second phase of the AREFD project. Ten individual interviews and four focus groups were conducted. Twelve males and ten females took part. Seven participants were Catholic clergy or in a Catholic religious order with the remaining fifteen identifying as lay Catholics. Contexts in which they have worked included retreat centres, pilgrimage sites, Catholic school management, academic research in religious education, training for voluntary pastoral ministry, evangelical ministry, diocesan advisory services at primary and post-primary level, youth ministry, and parish ministry.

## Analysis

All interviews were transcribed by the post-doctoral researcher. Transcripts were anonymised using labels for participants (e.g. P1, P2) and focus groups (e.g. G1, G2). Manual thematic analysis was then conducted over a number of reviews to initially code for broad themes. Data extracts were chosen to illustrate the most prevalent and pertinent issues that were raised across a number of interviews. These were further reviewed to consolidate the key findings into distinct themes.

## Results, discussion and recommendations

This paper reports upon four themes that emerged from the analysis of the data gathered from the phase two interviews of the AREFD project and applies them to the context of ITE for religious educators, specifically at post-primary level in the Irish context. In each case, a small number of quotations from participants is included for illustrative purposes. It is proposed, that the learnings can be applied in other jurisdictions, educational settings and age-groups.

## Specific realm of AREFD

This is the core theme emerging from the data and it is from this that the remaining themes follow. The participants highlighted the lack of opportunities in religious education and faith development specifically for adults:

The reason that I and we have been involved in adult education is because nobody else was involved in it

(P15)

They love the opportunity to pray together because in many parishes there isn't a set program for adult faith development or programs

(P12)

On the flip side of this recognised lack of opportunity, was the acknowledged appetite and hunger, indeed the need, amongst adults for such opportunities:

we're starting to feel that there is this, you know, seeking, particularly whenever people reach a point in their life, where they are questioning what's going on, or they're maybe distant from maybe their local parish or church. And either that's through their own reason that they want to do that, but that there's something else missing

(P2)

it shows there is an appetite if you can get the pitch right at the right level.

(P22)

There are particular events, milestones, social, physical and cognitive development stages considered common and to some extent expected amongst the general population of children and young people. This helps educators to identify needs and create targeted learning experiences, largely through curriculum content. The participants in phase two of the AREFD project, however, highlighted the massive spectrum of adulthood, noting how adulthood is far less compactible into neat little boxes that might inform programmes, courses and initiatives:

[we need to] find out where people are at and try and cater for their educational and faith formation development needs at that particular level

(P9)

we must speak at a level that responds to a need in the person.

(P15)

in some ways when it comes down to like what's needed if adult faith development is to happen and you know, first of all, we have to discern where are there possibilities

(P8)

The common default position of relating education in general to formal domains such as programmes, courses and initiatives is also problematic for the adult sphere, according to the participants. The need of the adult in terms of their religious education and faith development, must come from the adult in his or her own agency as a unique individual in unique circumstances:

a listening process that would generate needs and they will be responding to those needs and in responding to those needs they will be training people to meet them

(P6)

This need must be articulated by the adult. There are two key problems with this. One is religious literacy. Most adults are stuck in the religious language of their youth from formal engagement in school-based RE and/or catechesis:

there is an appetite there, but people haven't got the language to say what it is they want

(P4)

The second issue is cultural. The clerical and hierarchical model of the church which has dominated for centuries has disempowered many adults, giving them the wrong sense that they have to meet some unspoken set of criteria in order to initiate some intentional AREFD in their lives. For example, if they are not engaged with church on a regular basis, who do they think they are to articulate a religious need? Are they (in their perception) ‘holy enough?’:

there's still a myth as well, isn't there, that you have to be a certain type of person to work in the church or to belong. Some people still think well I wouldn't be holy enough for that

(P12)

It is very difficult to break laypeople who are very often the first to volunteer in a parish, it is very hard to break them away from the notion of father and bishops.

(P10)

The breadth of adulthood, spanning decades if viewed chronologically and spanning depths if viewed in life experience, is vast. Applying what has been learned here to ITE, there can be a pressure upon religious educators, where there is a perception that school-based religious education might be the only religious education available to a person. Consequently, there can be a tendency to feel that one must pack a lifetime’s worth of religious education and faith development into this schooling-based timeframe. This of course is unachievable and can detract from the quality of the experience of the young person of their school-based religious education. Instead, it is recommended that initial teacher education explicitly articulates that the religious education offered to the school-going population is appropriate to their current age and life experience. ITE should promote the understanding that post-primary students should expect that their school-based religious education does not encapsulate the breadth of, or address their religious education and faith development needs for their entire lives. Instead, they should be encouraged to anticipate the need for further religious education and faith development opportunities that will be appropriate to their needs as they grow older. In addition, in promoting the vision of education as lifelong, ITE should encourage the educators of the future to empower their students to welcome the prospect of appropriate and specific ongoing adult religious education and faith development opportunities.

## Intentionality

The majority of participants explained their involvement with adults in religious education and faith development as engagements that prepare adults for, or assist them in, an already existing role. Most commonly, this is the role of parent, guardian, grandparent or someone involved in leadership with groups of young people:

we work with a lot of volunteers and that helps in developing adult faith there as well

(P11)

So a lot of what we do is trying to empower or train adults to work with the young people in youth ministry context

(P12)

The implicit goal of the AREFD described above is not therefore the development and education of the adults in their religious or faith capacities. Instead, any personal development or growth in faith or any deepening of religious education in the adult was more of an offshoot that may or may not (but hopefully would) happen:

you're just trying to nurture that so they can nurture their children. We have a very strong belief here that it's through the children that we will reach the adults

(P11)

the future of religious education was to turn the model of the church upside-down, that we get to the parents through the children because the children exist for us - the parents don't. We can't find them. A lot of them, you know, because they live in different spheres to us, whereas the children, we see them in the school

(P15)

Participants generally highlighted the lack of focus on the identity of the adult as just that—the adult. That is to say not the adult in a role, such as parent, teacher, minister, neighbour, citizen because roles change over our lifetimes. Instead, the participants commented upon the dangers of perceiving the target of engaging in AREFD with the adult ‘in role’ rather than the adult as person, than we risk missing the deep encounters and experiences:

they're facing a lot of big questions and they don't need people to give them the answers, but they need a space to acknowledge what they've been through

(P17)

So if somebody is going to take something and take it just the bare bones, they don't have the depth of experience

(P17)

For initial teacher education then, it is important that the student teacher be acknowledged as an adult in his/her own right and not just in role as student teacher. When these adults are then working as religious educators in schools, they may be more inclined to, in turn, see their students and the other members of the school community as individuals in their own right rather than in the role of student, colleague, teacher and so on. To facilitate this vision, it would be of benefit for ITE to offer the opportunity of intentional AREFD for their student teachers, both within and beyond the prescribed course content.

## Physicality of religion

There was a great sense of energy and emotional investment amongst the participants in phase two of the AREFD project. This was something that they felt that they gained from involvement in AREFD that was also characteristic of their own contribution to their work.

it's something that has really inspired me, informed me, motivated me.

(P9)

I am driven. I am passionate and I have a real belief that the impossible is possible. You just have to find the way of doing it

(P15)

There was an animation evident amongst the participants when they implied their involvement in AREFD impacted upon their physical and mental health, contributing to feelings of enthusiasm, positivity and creativity. It was more than just the knowledge about religion, education and/or faith, but a more holistic and embodied experience where they were fully engaged in ‘head, heart and hands’:

if we say we believe this and these are our beliefs and these are our values, how do we make that tangible? So it's through our behaviours and actions.

(P4)

The importance of the physicality of religion was named by one particular participant as crucial:

Our religion is physical. It's actually a physical religion that is engaged with the senses. It's not... if we keep it in our heads, it becomes a political discussion and you opt in and you opt out

(P15)

And until we see our God as physical, it's very hard then to reject and not to engage.

(P15)

This participant continued to caution against not embracing the physicality of religion, particularly when engaging in religious education and faith development activities as an educator:

How did we come to that point? I think it was when we stopped trusting the heart and went to the head and we thought if we give them the knowledge, they'll know what to do. And therefore, we were enabling them not to have to think. And that's safer. And it's much better if we want to control. And we all do want to control.

(P15)

Another aspect of the physicality of religion is that of the sensorial experience. A number of participants highlighted the importance of engaging the senses:

We need to make our offering more hospitable to their senses.

(P5)

every part of their body is engaged, mental, emotion, physical

(P2)

The sensorial experiences in religious education have rightly been given more attention in recent years for students, both in primary and post-primary schools, but also, at the level of ITE. An example is visiting places of worship, especially belonging to different faiths. The physical experience of standing in a sacred space, listening to music of a faith community, witnessing or partaking in rituals or liturgies with many different sights, sounds and smells, can add a depth of understanding and a profound respect for one’s own faith and the faith or worldview of others. It is to be hoped that such broad appreciation continues to be cultivated in ITE for the aspects of religious education and faith development beyond, though including, the ‘knowledge-based’ features. This applies to the experience of the student RE teacher in their ITE and continuing professional development as well as the methodologies employed in the currriculums and learning experiences for which the student teachers are being prepared.

## Collaboration, communication and connection

The participants noted a pitfall in the practices of those involved in AREFD, namely the lack of communication and sharing of resources between various individuals and groups across the country and in different contexts. They noted how different groups worked largely in isolation from one another although at a basic level each was conducting similar activities:

without kind of joined up thinking, without investment, it's very hard to see where we are, where it's going

(P14)

A particular danger of this, as noted by the participants, was the consequences borne by personnel carrying out work that could be shared more broadly:

But unless you get people who are committed locally on the ground. Yes. So it's really difficult. And we've been so lucky. I mean, there are so many volunteers who do that. And unfortunately, it ends up as in many things, you probably know this from your own work. A small core of people take responsibility and then they get, you know, overworked.

(P21)

There was also, amongst the participants, an awareness of a lack of reflective practice. Instead, there was a trend to continue creating resources, conducting activities and when that initiative was complete, to move onto the next one:

It's funny to sit down to talk, because we never do this.

(P13)

We've never talked like this. We just work

(P12)

Initiatives often followed one after another, without a real sense of evaluating the strengths and weakness of an approach. As a result, the same obstacles were encountered time after time:

we kept doing the same thing

(P14)

In some cases, there was no deep investigation that could help towards explaining a falloff in interest and lack of long-term engagement amongst adults who took part in some way in AREFD activities, either as participant or provider. The positive outcomes of reflective practice were, nonetheless, appreciated:

one of the benefits of self-evaluation is to actually audit where you are at a given period, a given point in time. That's a snapshot of where you are and what you're doing.

(P3)

Related to this inertia that can emerge from a lack of reflective practice was the expressed difficulty amongst the participants in finding people who could take over from those in positions of leadership or continue the work in some way:

And if they're stuck in that model, people with new energies won't join because they'll only see the same old, same old dead stuff going nowhere fast.

(P10)

As the AREFD project is housed in a research centre of a university that provides ITE across the spectrum of early childhood through the levels to further and higher education, the research team were aware, first hand, of the benefits of sharing expertise across the different fields. This awareness was supported by the data in phase two of the AREFD project. Participants promoted an understanding of how different ‘ministries’ were connected and how sharing resources, be that personnel, ideas or content, is mutually beneficial:

find it hard to justify why primary ministry would be different from secondary school. And why sacramental preparation is different from parish ministry and adult faith formation is different from young adults or youth ministry. I don't think there's strong justification to say all those ministries should be separate. We are all one.

(P12)

In relation to ITE, an interdisciplinary approach is therefore encouraged. This is an approach that welcomes developments in theory and methodology from other sectors, acknowledging the multi-faceted and lifelong nature of education. Reflective practice is also recommended. This is already a key feature in much of recent curricular reform whereby equipping students with life skills such as self-awareness and critical thinking is a core principle. Nonetheless, it is one that must be regularly and explicitly encouraged, as it is a natural occurrence that the busyness of a heavy workload can result in ploughing ahead without stopping to reflect and evaluate.

## Conclusion

The rich data collected from the consultations is testament to the breadth of experience of those interviewed. Their insights not only speak to the pastoral sphere, but also to issues of organisation, leadership, curriculum development, theological underpinnings and theories of education, all of which are pertinent in academia. This indicates that much learning can be achieved for those involved in adult religious education and faith development at both pastoral and academic levels. In keeping with a vision of religious education that is lifelong and relational, it is appropriate that learnings from research, such as those reported upon in this paper, be offered for benefit across educational sectors. The AREFD project brings to light aspects of adult education, religious education and faith development. All of these areas are important in the initial teacher education of religious educators. It is proposed that the findings outlined in this paper will be of benefit to those involved in ITE of religious educators as they reflect upon their work and to those participating in ITE as adults as they continue in their lifelong education.
